# Supplementary material for: Metagenomic and Untargeted Metabolomic Analysis of the Effect of Sporisorium reilianum Polysaccharide on Improving Obesity
Source: Foods. 2023 Apr 7;12(8):1578. doi: 10.3390/foods12081578 (PMC10137368; doi:10.3390/foods12081578)
Supplement: Supplementary file 1 [file foods-12-01578-s001.zip › Supplemental material-Table.docx]

Table S1 Raw data of metagenomic sequencing

| Samples | Insert Size (bp) | Read length (bp) | Raw reads | Raw base (bp) |
| --- | --- | --- | --- | --- |
| NC1 | 500 | 150 | 56495284 | 8530787884 |
| NC2 | 500 | 150 | 49902614 | 7535294714 |
| NC3 | 500 | 150 | 57028986 | 8611376886 |
| NC4 | 500 | 150 | 50299938 | 7595290638 |
| HFD1 | 500 | 150 | 47058014 | 7105760114 |
| HFD2 | 500 | 150 | 51852678 | 7829754378 |
| HFD3 | 500 | 150 | 61631418 | 9306344118 |
| HFD4 | 500 | 150 | 48017450 | 7250634950 |
| HFD-SRP1 | 500 | 150 | 50048826 | 7557372726 |
| HFD-SRP2 | 500 | 150 | 50623944 | 7644215544 |
| HFD-SRP3 | 500 | 150 | 43451076 | 6561112476 |
| HFD-SRP4 | 500 | 150 | 52064864 | 7861794464 |

Table S2 Quality control data of metagenome sequencing

| Samples | Clean reads | Clean base(bp) | Percent in raw reads(%) | Percent in raw bases(%) |
| --- | --- | --- | --- | --- |
| NC1 | 55390120 | 8349321842 | 98.04379424 | 97.87281029 |
| NC2 | 48879760 | 7366788351 | 97.95029976 | 97.7637721 |
| NC3 | 55847244 | 8416110803 | 97.92782218 | 97.73246386 |
| NC4 | 48786072 | 7353873796 | 96.99032233 | 96.82149303 |
| HFD1 | 46138332 | 6955545509 | 98.04564213 | 97.88601638 |
| HFD2 | 50834408 | 7664099230 | 98.03622486 | 97.88428679 |
| HFD3 | 60419010 | 9108188730 | 98.03280853 | 97.8707494 |
| HFD4 | 47007406 | 7088059936 | 97.89650637 | 97.75778239 |
| HFD-SRP1 | 48734818 | 7346261038 | 97.37454781 | 97.20654657 |
| HFD-SRP2 | 49647278 | 7484667084 | 98.07074297 | 97.91282102 |
| HFD-SRP3 | 42424514 | 6393304633 | 97.6374302 | 97.44238734 |
| HFD-SRP4 | 50825660 | 7663624673 | 97.6198843 | 97.47933132 |

Table S3 Metagenomic sequencing gene prediction

| Sample | ORFs | Total Length(bp) | Average Length(bp) | Max(bp) | Min(bp) |
| --- | --- | --- | --- | --- | --- |
| NC1 | 641449 | 362471496 | 565.08 | 111252 | 102 |
| NC2 | 450039 | 251388726 | 558.59 | 27687 | 102 |
| NC3 | 628132 | 358965474 | 571.48 | 80841 | 102 |
| NC4 | 364325 | 202734132 | 556.47 | 44949 | 102 |
| HFD1 | 615709 | 303673914 | 493.21 | 13047 | 102 |
| HFD2 | 476515 | 271508745 | 569.78 | 21087 | 102 |
| HFD3 | 512918 | 268627689 | 523.72 | 20556 | 102 |
| HFD4 | 653392 | 327544449 | 501.3 | 14604 | 102 |
| HFD-SRP1 | 634379 | 311383896 | 490.85 | 21552 | 102 |
| HFD-SRP2 | 557663 | 292495479 | 524.5 | 21552 | 102 |
| HFD-SRP3 | 522672 | 273267804 | 522.83 | 17271 | 102 |
| HFD-SRP4 | 710713 | 363085995 | 510.88 | 14916 | 102 |

Table S4 Identification of metabolites in the feces samples of rat（Positive and negative ion mode）

| SN | Common name | Adducts | Retention time | m/z | Formula | HMDB | HFD/NC FC and trend | | HFD-SRPH/HFD FC and trend | |
| --- | --- | --- | --- | --- | --- | --- | --- | --- | --- | --- |
| C1 | myo-Inositol | M+H | 0.477 | 181.1638 | C_6_H_12_O_6_ | HMDB0000211 | 0.08 | ↓ | 1.35 | ↑ |
| C2 | Creatine | M+H | 0.65 | 132.1019 | C_4_H_9_N_3_O_2_ | HMDB00064 | 1.11 | ↑ | 0.98 | ↓ |
| C3 | Alanyltryptophan | M+H | 0.665 | 276.1311 | C_14_H_17_N_3_O_3_ | HMDB0013209 | 0.76 | ↓ | 1.44 | ↑ |
| C4 | L-Phenylalanine | M+H | 0.94 | 166.0858 | C_9_H_11_NO_2_ | HMDB0000159 | 1.20 | ↑ | 0.75 | ↓ |
| C5 | Lithocholic acid glycine conjugate | M+H | 3.378 | 434.6253 | C_26_H_43_NO_4_ | HMDB0000698 | 0.50 | ↓ | 0.27 | ↓ |
| C6 | Oxalosuccinic acid | M+NH4 | 4.104 | 208.0405 | C_6_H_6_O_7_ | HMDB0003974 | 0.04 | ↓ | 1.14 | ↑ |
| C7 | 5-Hydroxyindoleacetic acid | M+H | 4.429 | 192.0654 | C_10_H_9_NO_3_ | HMDB0000763 | 0.49 | ↓ | 0.42 | ↓ |
| C8 | Cyclic AMP | M+H | 4.689 | 330.2138 | C_10_H_12_N_5_O_6_P | HMDB0000058 | 2.25 | ↑ | 0.91 | ↓ |
| C9 | L-Tryptophan | M+H | 5.847 | 205.0858 | C_11_H_12_N_2_O_2_ | HMDB0000929 | 0.24 | ↓ | 0.85 | ↓ |
| C10 | LysoPC(14:0/0:0) | M+H | 6.136 | 468.3054 | C_22_H_46_NO_7_P | HMDB0010379 | 5.91 | ↑ | 0.88 | ↓ |
| C11 | Cholic acid | M-H | 6.707 | 431.2116 | C_24_H_40_O_5_ | HMDB0000619 | 0.14 | ↓ | 33.56 | ↑ |
| C12 | Urobilin | M+H | 6.723 | 591.3416 | C_33_H_42_N_4_O_6_ | HMDB0004160 | 0.71 | ↓ | 0.90 | ↓ |
| C13 | Arachidonic acid | M+H | 6.942 | 305.4702 | C_20_H_32_O_2_ | HMDB0001043 | 0.57 | ↓ | 0.72 | ↓ |
| C14 | Lithocholic acid | M+H | 7.105 | 377.2834 | C_24_H_40_O_3_ | HMDB0000761 | 2.95 | ↑ | 0.72 | ↓ |
| C15 | Palmitic acid | M+H | 7.541 | 257.2252 | C_16_H_32_O_2_ | HMDB0000220 | 0.04 | ↓ | 3.44 | ↑ |
| C16 | linoleic acid | M-H | 7.577 | 279.2309 | C_18_H_32_O_2_ | HMDB0000673 | 0.55 | ↓ | 1.31 | ↑ |
| C17 | Glycocholic acid | M-H | 7.88 | 464.3013 | C_26_H_43_NO_6_ | HMDB0000138 | 4.56 | ↑ | 0.66 | ↓ |
| C18 | Docosahexaenoic acid | M+H | 8.905 | 329.3659 | C_22_H_32_O_2_ | HMDB0002183 | 0.85 | ↓ | 1.31 | ↑ |
| C19 | Stercobilinogen | M+H | 8.999 | 597.3632 | C_33_H_48_N_4_O_6_ | HMDB0004157 | 1.00 | ↑ | 0.69 | ↓ |
| C20 | 12,13-DHOME | M+H | 9.209 | 315.2524 | C_18_H_34_O_4_ | HMDB0004705 | 0.08 | ↓ | 0.70 | ↓ |
| C21 | Sphingosine | M+H | 9.227 | 300.2893 | C_18_H_37_NO_2_ | HMDB0000252 | 10.15 | ↑ | 1.07 | ↑ |
| C22 | Sphinganine | M+H | 9.381 | 302.3046 | C_18_H_39_NO_2_ | HMDB0000269 | 1.61 | ↑ | 1.84 | ↑ |
| C23 | Isodeoxycholic acid | M-H | 9.395 | 391.2866 | C_24_H_40_O_4_ | HMDB0002536 | 0.09 | ↓ | 1.81 | ↑ |
| C24 | 13-HODE | M+H | 9.807 | 297.2418 | C_18_H_32_O_3_ | HMDB0004667 | 1.19 | ↑ | 1.09 | ↑ |
| C25 | LysoPC(18:2(9Z,12Z)/0:0) | M+H | 10.498 | 520.3388 | C_26_H_50_NO_7_P | HMDB0010386 | 0.67 | ↓ | 0.56 | ↓ |
| C26 | LysoPC(16:0/0:0) | M+H | 10.881 | 496.3378 | C_24_H_50_NO_7_P | HMDB0010382 | 0.28 | ↓ | 1.16 | ↑ |
| C27 | Chenodeoxycholic acid | M+H | 10.953 | 393.5798 | C_24_H_40_O_4_ | HMDB0000518 | 2.00 | ↑ | 0.66 | ↓ |
| C28 | LysoPC(18:1(9Z)/0:0) | M+H | 11.179 | 522.3543 | C_26_H_52_NO_7_P | HMDB0002815 | 0.44 | ↓ | 1.25 | ↑ |
| C29 | 2-Hydroxystearic acid | M+H | 11.772 | 301.2729 | C_18_H_36_O_3_ | HMDB0062549 | 0.72 | ↓ | 0.92 | ↓ |
| C30 | LysoPC(18:0/0:0) | M+H | 12.059 | 524.37 | C_26_H_54_NO_7_P | HMDB0010384 | 0.49 | ↓ | 1.07 | ↑ |
| C31 | alpha-Dimorphecolic acid | M+H | 12.404 | 297.2416 | C_18_H_32_O_3_ | HMDB0004670 | 0.67 | ↓ | 9.46 | ↑ |
| C32 | LysoPC(17:0/0:0) | M+H | 12.408 | 510.4494 | C_25_H_52_NO_7_P | HMDB0012108 | 0.82 | ↓ | 2.38 | ↑ |
| C33 | Eicosapentaenoic acid | M-H | 12.916 | 301.2194 | C_20_H_30_O_2_ | HMDB0001999 | 0.13 | ↓ | 0.89 | ↓ |
| C34 | Adrenic acid | M-H | 14.381 | 331.2665 | C_22_H_36_O_2_ | HMDB0002226 | 0.18 | ↓ | 1.44 | ↑ |
| C35 | Elaidic acid | M-H | 14.481 | 281.2503 | C_18_H_34_O_2_ | HMDB0000573 | 0.59 | ↓ | 1.03 | ↑ |
| C36 | Deoxycholic acid | M+H | 14.783 | 393.5786 | C_24_H_40_O_4_ | HMDB0000626 | 0.35 | ↓ | 3.93 | ↑ |

Table S5 Metabolic pathways enrichment table of feces samples

| Pathway Name | Match Status | p | -log(p) | Holm p | FDR | Impact |
| --- | --- | --- | --- | --- | --- | --- |
| Biosynthesis of unsaturated fatty acids | 5/36 | 0.00 | 2.41 | 0.33 | 0.33 | 0.00 |
| Sphingolipid metabolism | 3/21 | 0.02 | 1.62 | 1.00 | 1.00 | 0.20 |
| Phenylalanine, tyrosine and tryptophan biosynthesis | 1/4 | 0.12 | 0.93 | 1.00 | 1.00 | 0.50 |
| Linoleic acid metabolism | 1/5 | 0.14 | 0.84 | 1.00 | 1.00 | 1.00 |
| Primary bile acid biosynthesis | 3/46 | 0.16 | 0.79 | 1.00 | 1.00 | 0.02 |
| Ascorbate and aldarate metabolism | 1/10 | 0.27 | 0.57 | 1.00 | 1.00 | 0.00 |
| Phenylalanine metabolism | 1/12 | 0.31 | 0.51 | 1.00 | 1.00 | 0.36 |
| Fatty acid degradation | 2/39 | 0.34 | 0.47 | 1.00 | 1.00 | 0.00 |
| Tryptophan metabolism | 2/41 | 0.36 | 0.45 | 1.00 | 1.00 | 0.16 |
| Aminoacyl-tRNA biosynthesis | 2/48 | 0.43 | 0.36 | 1.00 | 1.00 | 0.00 |
| Citrate cycle (TCA cycle) | 1/20 | 0.46 | 0.33 | 1.00 | 1.00 | 0.00 |
| Galactose metabolism | 1/27 | 0.57 | 0.24 | 1.00 | 1.00 | 0.00 |
| Phosphatidylinositol signaling system | 1/28 | 0.58 | 0.23 | 1.00 | 1.00 | 0.04 |
| Purine metabolism | 2/66 | 0.61 | 0.22 | 1.00 | 1.00 | 0.00 |
| Inositol phosphate metabolism | 1/30 | 0.61 | 0.22 | 1.00 | 1.00 | 0.13 |
| Glycine, serine and threonine metabolism | 1/34 | 0.66 | 0.18 | 1.00 | 1.00 | 0.00 |
| Glycerophospholipid metabolism | 1/36 | 0.68 | 0.17 | 1.00 | 1.00 | 0.02 |
| Arachidonic acid metabolism | 1/36 | 0.68 | 0.17 | 1.00 | 1.00 | 0.33 |
| Arginine and proline metabolism | 1/38 | 0.70 | 0.16 | 1.00 | 1.00 | 0.01 |
| Fatty acid elongation | 1/39 | 0.71 | 0.15 | 1.00 | 1.00 | 0.00 |
| Fatty acid biosynthesis | 1/47 | 0.77 | 0.11 | 1.00 | 1.00 | 0.01 |

Table S6: *p* value of correlation between intestinal microbial species and differential metabolites

|  | Lactobacillus_crispatus | Lactobacillus_reuteri | Staphylococcus_xylosus | Lactobacillus_helveticus | Lactobacillus_amylovorus | Lactobacillus_acidophilus | Bacteroides_sp._CAG_927 | Barnesiella_sp._WM24 | Clostridium_sp._CAG_678 | Prevotella_sp._CAG_485 | Prevotella_sp._CAG_873 |
| --- | --- | --- | --- | --- | --- | --- | --- | --- | --- | --- | --- |
| C1 | 0.00736 | 0.05859 | 0.04461 | 0.03068 | 0.05859 | 0.02203 | 0.04786 | 0.07089 | 0.06251 | 0.02019 | 0.05859 |
| C2 | 0.77873 | 0.31914 | 0.35415 | 0.86290 | 0.74561 | 0.91409 | 0.44326 | 0.39110 | 0.63309 | 0.60210 | 0.18256 |
| C3 | 0.11211 | 0.15911 | 0.10060 | 0.24527 | 0.40383 | 0.27557 | 0.05859 | 0.03581 | 0.00920 | 0.05127 | 0.08487 |
| C4 | 0.36625 | 0.05859 | 0.29690 | 0.89699 | 0.79541 | 0.64880 | 0.60210 | 0.69668 | 0.37857 | 0.84593 | 0.22621 |
| C6 | 0.00824 | 0.00001 | 0.07089 | 0.07089 | 0.09516 | 0.04461 | 0.08991 | 0.10060 | 0.05127 | 0.05859 | 0.07089 |
| C8 | 0.02203 | 0.00259 | 0.04461 | 0.03581 | 0.13766 | 0.05859 | 0.03859 | 0.03068 | 0.00512 | 0.02203 | 0.11211 |
| C10 | 0.01259 | 0.00395 | 0.01391 | 0.03859 | 0.08487 | 0.05127 | 0.02203 | 0.02203 | 0.01137 | 0.01391 | 0.03317 |
| C11 | 0.98279 | 0.49856 | 0.47053 | 0.60210 | 0.37857 | 0.55674 | 0.63309 | 0.49856 | 0.22621 | 0.84593 | 0.40383 |
| C14 | 0.03581 | 0.05484 | 0.07089 | 0.26524 | 0.21702 | 0.15911 | 0.06251 | 0.06661 | 0.02400 | 0.07536 | 0.01845 |
| C15 | 0.61752 | 0.66463 | 0.41677 | 0.58682 | 0.52730 | 0.58682 | 0.84593 | 0.96559 | 0.72919 | 0.94840 | 0.77873 |
| C16 | 0.58682 | 0.76212 | 0.13095 | 0.48445 | 0.60210 | 0.63309 | 0.07089 | 0.05859 | 0.26524 | 0.11211 | 0.08002 |
| C17 | 0.00259 | 0.03068 | 0.00114 | 0.01845 | 0.02400 | 0.01137 | 0.00033 | 0.00079 | 0.04786 | 0.00222 | 0.00001 |
| C18 | 0.07536 | 0.05859 | 0.01025 | 0.18256 | 0.31914 | 0.22621 | 0.05484 | 0.05859 | 0.01137 | 0.08002 | 0.04461 |
| C19 | 0.00161 | 0.05127 | 0.00042 | 0.00190 | 0.02610 | 0.00736 | 0.00033 | 0.00008 | 0.06251 | 0.00161 | 0.00259 |
| C23 | 0.08991 | 0.10060 | 0.34226 | 0.18256 | 0.22621 | 0.13766 | 0.34226 | 0.41677 | 0.57170 | 0.13766 | 0.33059 |
| C26 | 0.82902 | 0.51284 | 0.60210 | 0.57170 | 0.48445 | 0.61752 | 0.33059 | 0.33059 | 0.76212 | 0.66463 | 0.41677 |
| C27 | 0.00736 | 0.10060 | 0.00299 | 0.00451 | 0.01683 | 0.01259 | 0.00011 | 0.00006 | 0.02203 | 0.00004 | 0.00259 |
| C28 | 0.93123 | 0.93123 | 0.39110 | 0.84593 | 0.87992 | 0.93123 | 0.39110 | 0.30791 | 0.84593 | 0.77873 | 0.35415 |
| C30 | 0.89699 | 0.72919 | 0.52730 | 0.57170 | 0.61752 | 0.74561 | 0.76212 | 0.74561 | 0.61752 | 0.69668 | 0.28612 |
| C31 | 0.13766 | 0.13095 | 0.04461 | 0.26524 | 0.39110 | 0.27557 | 0.03859 | 0.03859 | 0.00299 | 0.05859 | 0.03581 |
| C32 | 0.13095 | 0.08991 | 0.04152 | 0.27557 | 0.55674 | 0.35415 | 0.03581 | 0.01683 | 0.07536 | 0.04461 | 0.02832 |
| C34 | 0.15174 | 0.10625 | 0.51284 | 0.24527 | 0.31914 | 0.22621 | 0.57170 | 0.66463 | 0.22621 | 0.25514 | 0.72919 |
| C35 | 0.26524 | 0.42992 | 0.84593 | 0.29690 | 0.26524 | 0.22621 | 0.94840 | 0.86290 | 0.91409 | 0.69668 | 0.69668 |
| C36 | 0.30791 | 0.76212 | 0.47053 | 0.23562 | 0.42992 | 0.34226 | 0.33059 | 0.24527 | 0.03859 | 0.24527 | 0.79541 |
