# Supplementary figures and images for: Metagenomic and Untargeted Metabolomic Analysis of the Effect of Sporisorium reilianum Polysaccharide on Improving Obesity
Source: Foods. 2023 Apr 7;12(8):1578. doi: 10.3390/foods12081578 (PMC10137368; doi:10.3390/foods12081578)

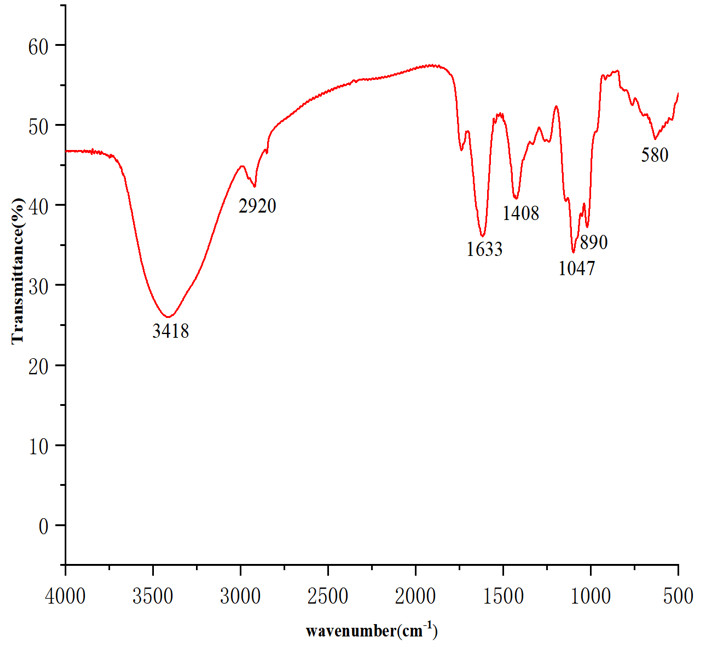


Figure S1 IR spectra of *Sporisorium reilianum* polysaccharide

Supplement: Supplementary file 1 [file foods-12-01578-s001.zip › Supplemental material-Figure.docx]
